# Does resistance training improve body image satisfaction among the elderly? A cross-sectional study

**DOI:** 10.6061/clinics/2018/e290

**Published:** 2018-07-10

**Authors:** Roberta Luksevicius Rica, Eliane Florencio Gama, Alexandre Fernandes Machado, Angélica Castilho Alonso, Alexandre L. Evangelista, Aylton Figueira-Junior, Marcelo Zanetti, Regina Brandão, Maria Luiza de Jesus Miranda, José Vilaça Alves, Marco Bergamin, Danilo Sales Bocalini

**Affiliations:** I Departamento de Educacao Fisica e Ciencias do Envelhecimento, Laboratorio de Percepcao Corporal e Movimento, Universidade Sao Judas Tadeu, Sao Paulo, SP, BR; II Divisao de Education Fisica, Departamento de Educacao, Universidade Nove de Julho, Sao Paulo, SP, BR; III Sport Sciences Department, University of Trás-os-Montes e Alto Douro, Vila Real, Portugal; IV Research Center in Sports Sciences, Health Sciences and Human Development, Vila Real, Portugal; V Sport and Exercise Medicine Division, Department of Medicine, University of Padova, Padova, Italy; VI Laboratorio de Fisiologia e Bioquimica Experimental, Centro de Educacao Fisica e Deportos, Universidade Federal do Espirito Santo (UFES), Vitoria, ES, BR

**Keywords:** Body Image, Aging, Resistance Training, Older Women

## Abstract

**OBJECTIVE:**

This study aims to evaluate whether body image satisfaction improves with practice in resistance training in elderly women.

**METHODS:**

Forty women were selected and randomly divided into an untrained group (UN) group and a group trained in resistance exercises (RT). To evaluate body image satisfaction, the silhouette matching task was used.

**RESULTS:**

No differences were found between current (5.45±0.24) and ideal (4.7±0.12) silhouettes in the RT group. However, the UN group showed a significant difference (*p*<0.01) between current (10.4±0.43) and ideal (5.6±0.26) silhouettes. The current silhouette was significantly different between the UN and RT groups (*p*<0.002). However, the ideal silhouette value did not differ between them. Body satisfaction was present in 90% of the participants in the RT group, and the difference was 15% less than that in the UN group (x^2^ test, *p*<0.001).

**CONCLUSION:**

Resistance training in elderly women can promote satisfaction with their body image.

## INTRODUCTION

Paul Schilder addressed the concept of body image (BI) in 1935, noting that every person has an image of him/herself that comprises biological, sociological and libidinal reality 1. This portrait is how we see our bodies, including information arising from touch, smell, nerves, muscles, viscera and thermal and pain sensations. Body perception is based on BI and body schema. Body schema is the representation of different body segments in space, which is essential to guide movement, and it is unconscious. As a conscious visual representation of the body, BI can be influenced by the environment, other people, desire and emotions 2,3.

In elderly people, the relationship between body image satisfaction and aging remains unclear 4. Considering that older subjects are vulnerable to negative experiences, an undesirable BI can be harmful for the aging process 5,6. Therefore, it is important to identify interventions that can improve BI in elderly people.

Physical exercise has been considered a key element of health promotion and improvement of overall physical fitness as well as an approach to increase body self-knowledge, self-esteem, self-perception and effectiveness, which are important factors for a positive BI 7-10.

The American College of Sports Medicine 11 recommends a regular physical exercise program for adults, including elderly people, with resistance exercises to improve muscle strength and functional capacity. However, there is little evidence 12 for a positive role of resistance training in BI; therefore, to fill this knowledge gap, this study aimed to evaluate whether resistance training (RT) can promote body satisfaction among elderly women.

## MATERIAL AND METHODS

### Subjects

Fifty volunteers (women over 60 years old) were recruited for this study. All participants had undergone medical screening and completed questionnaires regarding their medical history. The inclusion criteria were age >60 years old and no use of hormone replacement therapy, drugs and vitamins that could alter calcium or bone metabolism. Participants were excluded from participation in the study if they had any of the following conditions: participation in current or previous regular RT in the last 6 months (only for the control group), recent hospitalization, symptomatic cardiorespiratory disease, hypertension or metabolic disorders, severe renal or hepatic disease, cognitive impairment or progressive and debilitating conditions, marked obesity with inability to exercise, and recent bone fractures or any other medical contraindications to RT practice (for any group). According to the exclusion criteria, 10 subjects were excluded, and the remaining subjects were randomized to the untrained (UN; n=20) or resistance trained (RT; n=20) group.

### Physical exercise program approach

The program used, cited in a previous publication 6, was performed for six months and consisted of three one-hour sessions of resistance exercises on nonconsecutive days. The following resistance exercises were included: leg press, chest press, leg curl, latissimus pull down, elbow flexion, elbow extension, leg extension, upper back row, military press, hip abductor, hip adductor, and abdominal curls. All exercises were performed with RT-specific devices. The load and repetitions were three sets of 10 at 75% of 1RM for all exercises. Participants alternated exercises for the upper and lower muscle groups to minimize fatigue, with one minute of rest between them. For all exercises, eccentric muscle action was emphasized. All sessions were guided by trained fitness instructors and supervised by the researchers.

### Anthropometric parameters

Height (meters) was evaluated with a stadiometer (Cardiomed, WCS model; Paraná, Brazil) fixed to the wall and calibrated at a 0.1 cm scale. Body weight (kg) was assessed using an electronic scale (Filizzola Personal Line model 150; São Paulo, Brazil) accurate to 100 g. Body mass index (BMI) was calculated as follows: BMI = weight / (height)^2^.

### Functional strength tests

Senior tests were applied according to the validated recommendations from Rikli and Jones 13. The arm curl test and chair stand test were used to assess upper and lower functional strength. Each subject completed two familiarization trials before the test. The scores were the total number of curls through the full range of motion (arm curl test) and the total number of stands (chair stand test) executed correctly in 30 seconds.

### Body image satisfaction evaluation

**Figure 1:**
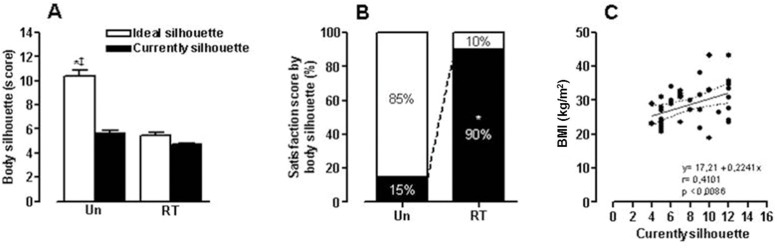
Panel A: values (mean±SEM) for the scores of current and ideal silhouettes of the UN and RT groups. ^‡^*p*<0.02 *versus* current silhouettes by the UN group. *p<0.01 versus ideal silhouettes by the RT group. Panel B: percentage of relative values about body satisfaction (black) and dissatisfaction (white) *p<0.001. Panel C: correlation between BI satisfaction and current BMI.

The instrument used to evaluate BI satisfaction was the silhouette matching task proposed by Stunkard, Sorenson and Schulsinger 14 and adapted by Marsh and Roche 15. This task consists of 12 silhouettes on a progressive scale (Figure 1). The discrepancy between the current (CS) and ideal silhouettes (IS) was analyzed after the figures were presented to the elderly women. The following questions were directed to the women: 1 What is the silhouette that best represents your current physical appearance? 2 What silhouette would you like to have?

### Statistical analyses

All analyses were performed using SPSS for Windows software (version 22 SPSS Inc., Chicago, Illinois, USA). All data are expressed as the mean±standard deviation. The D'Agostino-Pearson test was applied for Gaussian distribution analysis. The X2 test was used to identify category differences by frequency. A paired Student’s t-test was used to compare anthropometric and functional fitness parameters. Analysis of comparisons between currently and ideal silhouette were performed with two-way ANOVA followed by Bonferroni’s post hoc test. Analysis with Pearson correlations was conducted to assess the association between ideal silhouette and BMI. Statistical significance was established at *p*<0.05.

## RESULTS

The groups showed no significant differences in the anthropometric variables: height (UN: 1.60±1.2, RT: 1.60±1.6; m), weight (UN: 74±7 kg; RT: 72±7 kg), and BMI (UN: 29±5, RT: 28±4; kg/m2). The functional strength of the lower and upper limbs measured by the chair stand test (UN: 19±3, RT: 29±4; repetitions) and arm curl test (UN: 24±5, RT: 36±4; repetitions), respectively, was significantly higher in the RT group than in the UN group (*p*<0.001).

In relation to BMI (Figure 1A), no significant differences were found between the CS (5.45±0.24) and IS (4.7±0.12) in the RT group. However, the UN group showed a significant difference (*p*<0.01) between the CS (10.4±0.43) and IS (5.6± 0.26). Additionally, the CS result was significantly different (*p*<0.002) between the UN and RT groups. However, their IS values did not differ significantly, as shown in Figure 1B.

For BI satisfaction, significant differences were observed (X2, *p*<0.001). Eighteen women (90%) in the RT group and 17 (85%) in the UN group showed positive and negative satisfaction with their BI, respectively. Additionally, a positive correlation was observed (*p*<0.0086) between the BMI and CS parameters (Figure 1C).

## DISCUSSION

The main result of our study indicates the difference between the UN and RT groups as well as the similarity between the expected silhouettes in both groups. The data from this study were similar to those in Matsuo 16 in active elderly people, reflecting the contribution of physical activity to BI through increasing physical and functional fitness and improving self-perceptions of wellness (physiological, cognitive and emotional), thus improving self-esteem. These positive effects of physical activity can change the meaning of aging and body concept for elderly people and rebuild their BI with smaller discrepancies 9,10.

The greatest determinants for negative or positive BI are satisfaction with weight, accuracy of size perception, body satisfaction, appearance evaluation and orientation, body esteem, body ideal, body schema and body awareness 17. Moreover, BI can develop in relation to other factors, such as gender, age, media, and the relationship of the body with cognitive processes such as cultural and social beliefs, values and attitudes 17,18.

Human beings are biologically and socially dependent on their appearance: body form, skills, clothes, hair and appearance reflect integration and harmony with oneself 19. In a previous study, Ricciardell, McCabe and Banfield 20 found improvements in both self-image and self-esteem associated with physical activity. Additionally, several different physical changes were observed with exercise in the study by Ginis et al. 21, and these changes were related to different aspects of BI, such as sex, body composition and strength.

In the present study, the idealized BI of the UN group was thinner than what they considered to be realistic. This perception could be due to cultural variables reflecting societal beliefs largely influenced by the media in favor of a lean and perfect body with the appearance of youth. Moreover, this difference could be associated with the stereotype of old age: diseases and biological decline.

Additionally, we found a positive correlation between BMI and CS, supporting the use of mean BMI values grouped by actual profiles in the characterization of ideal physical types for elderly people.

A physically active lifestyle helps elderly women make their own judgments related to self-assessment and BI. Body dissatisfaction can affect their lives substantially; in Western culture, an older body is often viewed as unattractive and unproductive.

Some limitations should be considered in this study. The instrument used in this study assessed silhouettes bidimensionally and allowed for little individual identification. The distribution of subcutaneous fat and other anthropometric features also have an important role in BI 22,23, but these factors were not taken into account.

Our data indicate that resistance training can improve the BI satisfaction of elderly women. The positive evaluation of current silhouettes in the RT group may be related to their active lifestyles, thus supporting the use of physical activity to improve BI.

## AUTHOR CONTRIBUTIONS

Rica RL, Gama EF and Bocalini DS performed the laboratory procedures, designed the study and were the main contributors to the writing of the manuscript. Machado AF, Alonso AC and Figueira-Júnior A performed the laboratory procedures, as well as, recruiting the volunteers, conducting the anthropometric measurements and filling out the questionnaires. Zanetti M and Brandão R evaluated the data from the statistical analysis. The revision of the manuscript was performed by Evangelista AL, Alves JV and Bergamin M, who contributed to the intellectual content of the study.
